# Fishing out the signal in polypharmacological high-throughput screening data using novel navigator cheminformatics software

**DOI:** 10.1186/1758-2946-6-S1-P14

**Published:** 2014-03-11

**Authors:** Denis Fourches, Alexander Tropsha

**Affiliations:** 1 Laboratory for Molecular Modeling, Division of Chemical Biology and Medicinal Chemistry, UNC Eshelman School of Pharmacy, University of North Carolina at Chapel Hill, Chapel Hill, NC, 27599, USA

## 

Many drugs are characterized by polypharmacological mechanisms of action. Thus, prospective drug discovery studies often start by testing large compound libraries in multiple and diverse High-Throughput Screening (HTS) assays. These large heterogeneous data collections pose numerous computational challenges concerning processing, curation, and analysis of untreated output files generated by plate readers. We have developed the freely-accessible HTS Navigator software to enable and facilitate the processing and analysis of polypharmacological HTS data. We report on the capabilities of Navigator and present several case studies where we employed cheminformatics approaches embedded within the Navigator to curate and analyze large datasets of compounds tested toward different panels of targets. Examples include libraries of compounds tested for their inhibition potencies across several CYP450; or for their inhibition of multiple protein kinases; or their binding profiles against multiple GPCRs. We show how to quickly identify and highlight compounds with unique mono- and dual- selectivity for certain targets in the curated HTS matrix. We discuss the problem of experimental variability in HTS data and its consequences for molecular modeling and emphasize the synergistic potential of different cheminformatics approaches to detect both false-positive and false-negative compounds using neighborhood analysis and target baseline correction factors. Finally, we describe the Chemical−Biological Read-Across (CBRA) approach [1] also implemented in the Navigator to infer the activity of external compounds from both chemical (defined by chemical similarity) and biological (defined by the similarity of HTS profiles) analogues.
